# Developing an interprofessional identity complementary to a professional identity - findings related to Extended Professional Identity Theory (EPIT)

**DOI:** 10.3389/fmed.2024.1467362

**Published:** 2024-10-02

**Authors:** Jan Jaap Reinders, Mukadder İnci Başer Kolcu, Giray Kolcu

**Affiliations:** ^1^Research Group Interprofessional Education (IPE), LEARN, University Medical Center Groningen, University of Groningen, Groningen, Netherlands; ^2^Research Group Healthy Ageing Allied Health Care and Nursing, Hanze University of Applied Sciences, Groningen, Netherlands; ^3^Department of Behavioral and Community Dentistry, Center for Dentistry and Dental Hygiene, University Medical Center Groningen, University of Groningen, Groningen, Netherlands; ^4^Department of Medical Education and Informatics, Faculty of Medicine, Süleyman Demirel University, Isparta, Türkiye; ^5^Süleyman Demirel University Institute of Health Sciences, Isparta, Türkiye; ^6^Ege University Institute of Health Sciences PhD Candidate, İzmir, Türkiye

**Keywords:** interprofessional identity, EPIT, EPIs, IPE, IPECP, theory, interprofessional collaboration, motivation

## Abstract

Collaboration among various professions often faces barriers owing to divergent perspectives, priorities, and expertise shaped by distinct socialization processes. These differences can hinder effectiveness, efficiency, and workforce well-being. The Extended Professional Identity Theory (EPIT) addresses this issue by fostering an interprofessional identity without weakening professional identities. Drawing from psychological theories, EPIT explains the coexistence of interprofessional and professional identities, and predicts associated behaviors. It also emphasizes the importance of combining interprofessional identity formation with developing interprofessional competencies and adapting to environmental factors to achieve synergy in (temporary or permanent) mixed profession groups. Introduced in 2018, EPIT research initially relied on the measurement of congruent interprofessional behaviors as indirect indicators of interprofessional identity that could not yet be measured. An experiment demonstrated that enhancing social identification in mixed profession groups with interprofessional assignments reduced the social hierarchy within 6 h across three meetings. The 2020 development of the Extended Professional Identity Scale (EPIS) confirmed interprofessional identity as a three-dimensional social construct. So far, several scientific studies have supported many propositions of EPIT. These propositions are related to dimensionality and various psychometric properties, cross-cultural similarities, evidence and clues for interprofessional identity formation, and its predictive validity in interprofessional education and collaborative practice. Türkiye is among several countries (e.g., the Netherlands, Belgium, Germany, Lithuania, Finland, and Indonesia) where EPIT-based interprofessional identity is being investigated. To illustrate contextual differences and their potential cross-cultural implications, it is valuable to explore how interprofessional identity adds value in the Turkish context. This approach facilitates understanding the regional implications of interprofessional identity, including interprofessional education initiatives, increased university engagement, the development of measurement instruments, challenges and future directions, and national and international collaborations. This paper aims to explain and clarify EPIT propositions compared to other theories, describe current evidence, and outline future research directions, with a focus on developments within the Turkish context as a showcase.

## Introduction

Collaboration among members of various professions often encounters barriers due to differing perspectives, priorities, and expertise shaped by distinct socialization processes within their respective fields. However, interprofessional collaboration can overcome these challenges, leading to greater effectiveness (1, 2), increased efficiency (3, 4), and enhanced job satisfaction (5, 6).

Nurses and physicians, for instance, often have differing views on team communication (7), which can result in mutual criticism for perceived communication failures. Misunderstandings of roles and a lack of clear team leadership can negatively impact overall team performance. Similar role confusion exists among other professions, such as dentists and dental hygienists (8), occupational therapists, physical therapists, and physician assistants (9), as well as psychologists, exercise physiologists, and dietitians (10). Members of mixed profession groups may be reluctant to compromise their professional priorities or adapt solutions that significantly accommodate other professions, fearing it might diminish their professional distinctiveness (11). Moreover, a lack of shared expertise among various professions can hinder a more holistic approach to patient care (12).

The differing professional perspectives, priorities, and lack of shared expertise stem from separate socialization processes (13). Training within professional “social silos” leads to different frames of reference and social commitments (14). Yet, developing a profession-specific professional identity is essential as it is a source of motivation and, when the professional role is clear, guides behaviors and enhances performance (15). Distinct professional identities are also crucial for effective interprofessional collaboration (16). The diversity within mixed profession groups can be better utilized when members share a common team identity (17). However, this team identity is often linked to a specific team, and drastic changes in team composition can compromise shared identity and reduce group cohesion (18). In addition, such a shared identity would not have a transferable commitment to other teams, making this an inflexible social identity. This underscores the need for individuals to develop a shared identity related to interprofessional collaboration independent of a specific team identity.

Currently, three comprehensive theoretical frameworks have been developed to explain what interprofessional identity is and how it is formed. This paper describes one specific interprofessional identity theory: the Extended Professional Identity Theory (EPIT). EPIT is developed from a work and organizational psychological perspective and initially proposed for the Dutch oral healthcare (19). The purpose of this paper is to explain and clarify the propositions of EPIT in comparison with other theoretical approaches, describe current evidence, and outline future research directions, with a focus on development within the Turkish context as a showcase.

## Formation and activation of social identity: implications for interprofessional collaboration

Identity, according to a psychological perspective, refers to one’s sense of self shaped by individual personality, experiences and social interactions (20). This “self” is also known as “personal identity” which is distinct from a “social identity” (21–23). Social identity is linked to an individual’s psychological association with a group or a social category. Tajfel (24) defines social identity as “part of an individual’s self-concept which derives from his [or her] knowledge of his [or her] membership of a social group (or groups) together with the value and emotional significance attached to that membership.” This definition was tested by Cameron which resulted in the Three-Factor Model of Social Identity (25). This model was also validated by Obst and White (26). Psychological research on social identity has revealed different implications at intrapersonal and interpersonal levels.

Identity theory explains the intrapersonal level of social identity. This identity theory is one of two separate but complementary fields of psychological study regarding social identity (27). The second field of study concerns the social identity theory. Identity theory explains how individuals cope with their multiple social identities (28) while social identity theory explains intergroup processes in which a distinction is made between ingroup and outgroup members (29). The risk of competition or conflict between different professional groups is enhanced by their separate socialization processes. Misunderstanding, social hierarchy, and stereotyping result from divergent perspectives, priorities, and lack of clarity about roles and expertise (7–9, 11, 27).

**Figure 1:**
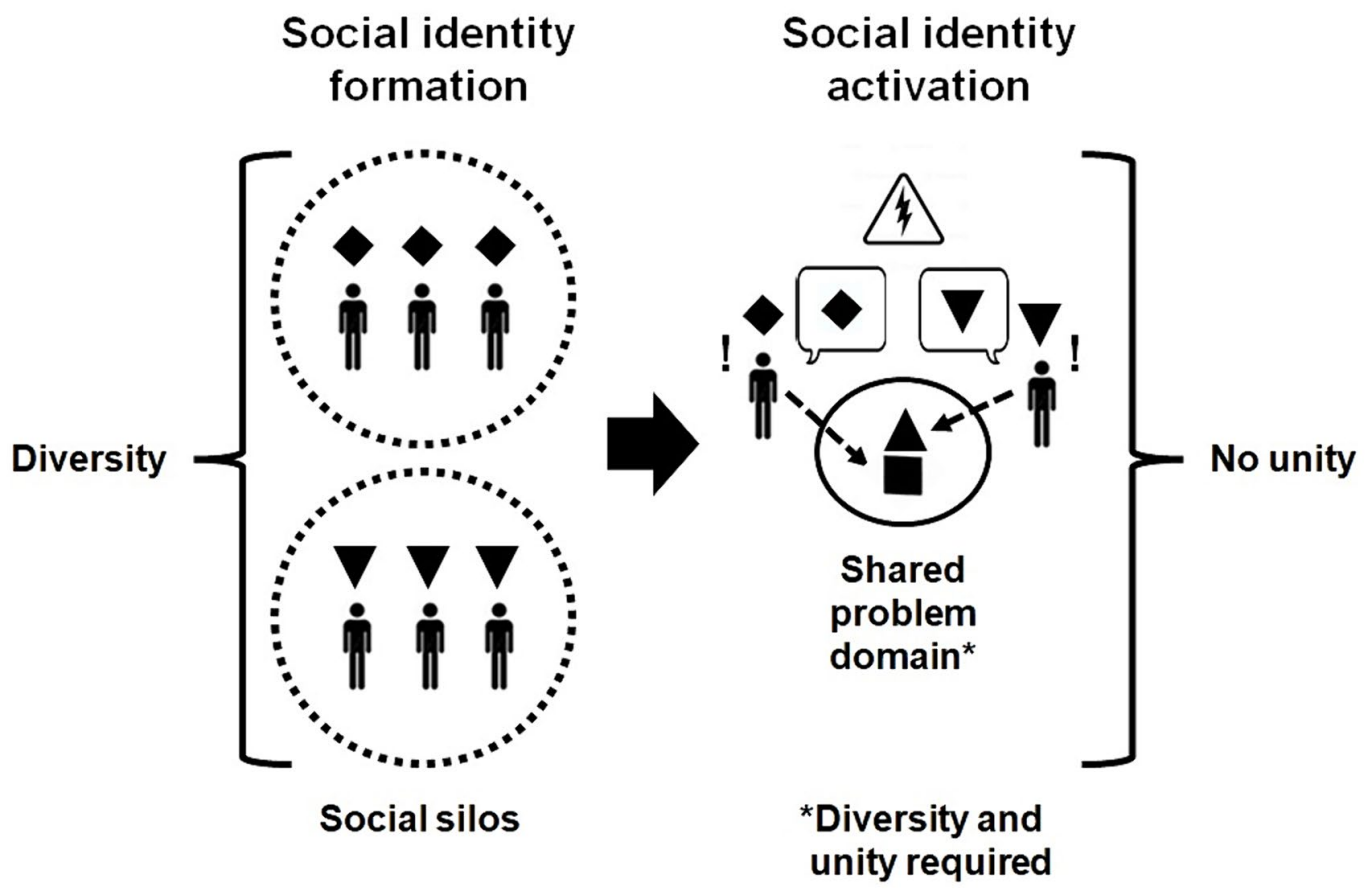
Isolated social identity formation versus activation in a mixed profession group.

Isolated group socialization results in the formation of unique social identities. Inherent in social identity formation is the individual internalization of role clarity as associated with the group concept and commitment to the group or social category. When individuals become members or anticipate future membership (anticipatory socialization) of a certain profession, each profession will have its own group composition of members, frame of reference, jargon, and competencies. Each individual (future) professional learns to interpret his or her professional environment. The subjective interpretation of this environment represents the “context” that is unique to the individual (future) professional. This means that an environment contains contextual cues that we have learned to recognize as important to one of our particular social identities of which each individual has many (28). Such contextual cues function as “identity triggers,” which activate the related social identity. In turn, this activation will lead to the display of congruent behaviors (27, 30). Therefore, the formation of a social identity, such as a professional identity, is essential and simultaneously a challenge when activated among individuals with different profession-specific professional identities (Figure 1). Thus, a professional identity fosters professional behaviors but does not necessarily promote interprofessional behaviors.

## Extended Professional Identity Theory and other interprofessional identity theories

**Figure 2:**
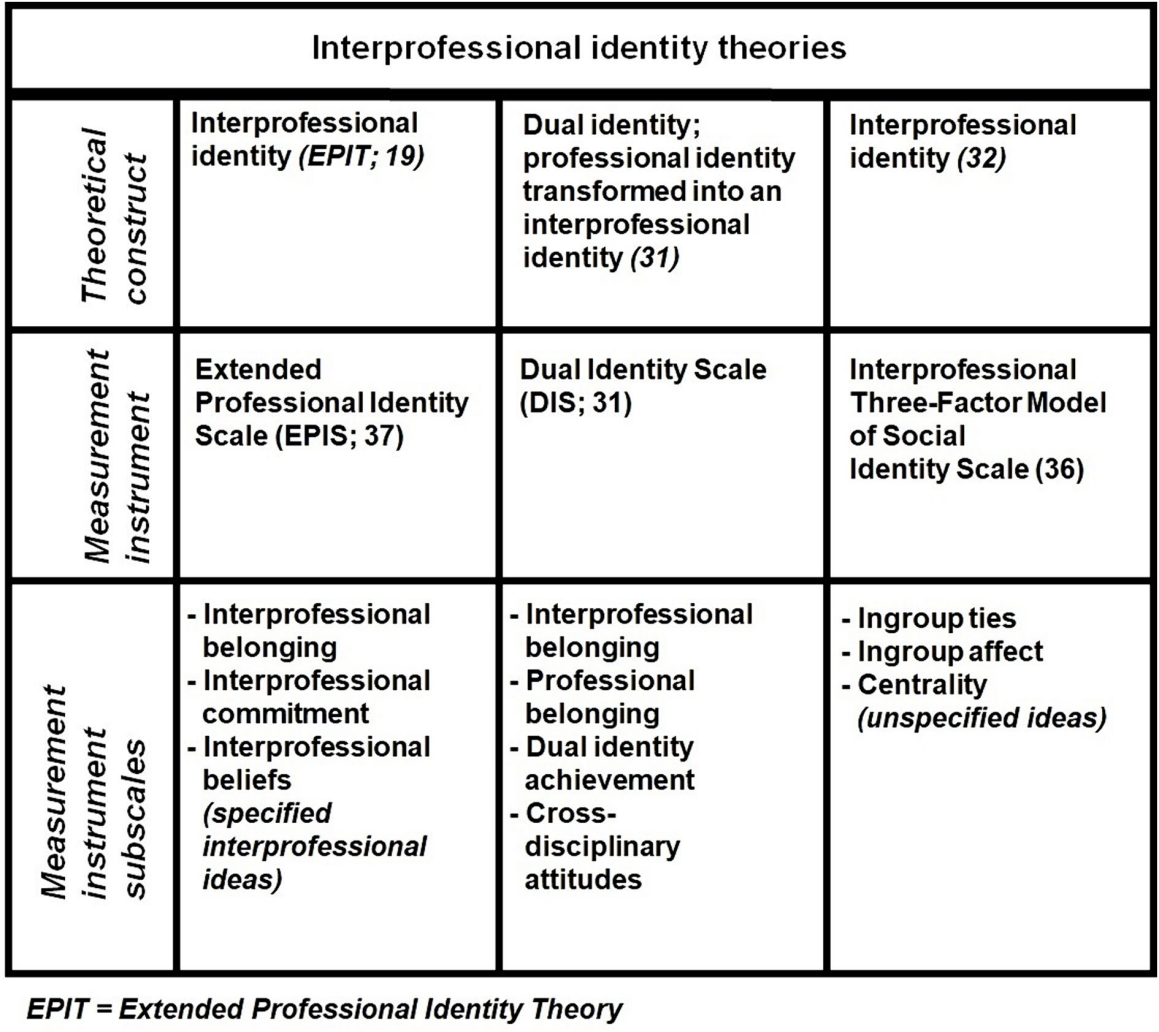
Comparison of interprofessional identity theories and their measurement instruments.

Four significant differences between interprofessional identity theories, comparing EPIT (19) with other theories (31, 32), are dimensionality, the role of attitudes, theory integration, and behavior prediction (Figure 2). Since each theory is (partially) based on different theoretical propositions, this affects the measurement of interprofessional identity. Given that these measurement instruments and their theoretical foundations differ, they cannot be used interchangeably, except for one or two similar subscales.

First, EPIT (19) and Tong’s theoretical approach (32) conceptualize interprofessional identity as a three-dimensional construct, while Khalili’s dual identity (31) consists of four dimensions (29). Tong’s (32) identity dimensions are derived from the work of Cameron (25), whereas EPIT’s (19) dimensions are based on psychological studies on affective commitment and other social identity factors but also aligns with the work of Cameron (25, 33–35). Tong’s theoretical approach applies Cameron’s work to measure interprofessional identity using her Interprofessional Three-Factor Model of Social Identity Scale (36). One of its three subscales, cognitive centrality, assesses how much individuals consider their membership in a mixed professional group or social category. This subscale measures the amount of time spent thinking about being a group member (25). These thoughts might involve interprofessional concepts, but could also include multiprofessional ideas, as this distinction is not explicitly defined. The Extended Professional Identity Scale (EPIS) (37) is based on EPIT (19) and is also three-dimensional but measures specific interprofessional beliefs (Table 1). It includes items such as “I like meeting and getting to know people from other health professions” (interprofessional belonging), “I would be very happy to spend the rest of my career with an interprofessional team” (interprofessional commitment), and “Joint clinical decision-making should be an important part of interprofessional collaboration” (interprofessional beliefs).

**Table 1:** Extended Professional Identity Scale (EPIS)—an interprofessional identity measure.

Subscale	Items
Interprofessional belonging	
	1. I like meeting and getting to know people from other health professions. 2. I feel a strong attachment toward interprofessional teams comprising cross-disciplinary health professionals. 3. I enjoy learning and collaborating with people from other health professions. 4. I like learning about other health professions.
Interprofessional commitment	
	5. I would be very happy to spend the rest of my career with an interprofessional team. 6. I identify myself with other members of an interprofessional team. 7. I am proud to be a part of an interprofessional team. 8. I prefer working with others in an interprofessional team.
Interprofessional beliefs	
	9. All members of an interprofessional team should be involved in goal setting for each patient. 10. When care decisions are made, the interprofessional team members should strive for consensus on planned processes. 11. Interprofessional team members should jointly agree to communicate plans for patient care. 12. Joint clinical decision-making should be an important part of interprofessional collaboration.

1 = strongly disagree; 2 = disagree; 3 = neutral/no opinion; 4 = agree; 5 = strongly agree.

Second, the roles of attitudes differ. Khalili (31) measures attitudes as an identity dimension, whereas EPIT (19) views attitudes as crucial antecedents to interprofessional identity formation (36), Attitudes, defined as positive or negative evaluations of objects, people, or events (37, 38), influence (affective) commitment and, thus, have motivational effects on the importance of a group to an individual (39).

Third, the integration of theories varies between interprofessional identity theories. Khalili’s dual identity (31) combines interprofessional belonging and professional belonging as identity dimensions of the same construct, while EPIT (19) and Tong (32) treat these two dimensions as dimensions of separate social identities, professional identity and interprofessional identity. EPIT (19), drawing from Turner’s Social Categorization Theory (40), posits that interprofessional identity is superordinate to professional identity. This framework acknowledges multiple social identities and the broader social categories individuals belong to (28). Unlike the dual identity approach (31), EPIT (19) states that attitude is an antecedent rather than an identity dimension, aligning with Allport’s Intergroup Contact Theory (41) which emphasizes the social process of reducing prejudice through active collaborative intergroup interactions creating positive attitudes towards members of different groups. Furthermore, EPIT (19) is the only theory that explicitly integrates identity theory and social identity theory as two separate but complementary psychological theories about social identity. This is in line with the work of Stets and Burke (27), who described how each individual has many social identities (identity theory) that can sometimes conflict with the social identities of other individuals (social identity theory). As a social identity is a source of motivation, each social identity serves an important purpose for groups and group memberships. When two social identities of one individual are simultaneously activated by their identity triggers, individuals do not have to choose between these identities if they are complementary. Identity mobility between professional identity and interprofessional identity can be triggered based on relevance of the perceived necessity of congruent actions in a certain context (42–44). This is also the reason why “extended” has been added to the name of the Extended Professional Identity Theory. This refers to extending the professional identity of an individual with an additional social identity. Other words for “extended” is “broadened” or “widened” which is in line with superordinate social identities as identification with “widening circles of group membership” (40). According to EPIT (19), interprofessional identity can be developed without altering professional identity, because both are separate and distinct social constructs. This assumption was tested by Bostedt et al. (45). If professional identity and interprofessional identity are distinct social identities, interprofessional socialization could be enhanced without changing professional identification. After interprofessional training, interprofessional socialization increased significantly with strong effect sizes while professional identity, measured with the Macleod Clark professional identity scale, indeed remained unchanged. This professional identity scale is not three-dimensional but does contain items related to professional belonging and professional commitment (46).

Fourth, behavior prediction differs among these theories. Effective measurement instruments should be able to predict relevant behaviors. The Interprofessional Three-Factor Model of Social Identity Scale (36) and EPIS (47) differ in that the former measures centrality, which reflects the amount of time spent thinking about group membership without necessarily involving normative interprofessional thoughts. Centrality is sufficient for demonstrating whether various social identities share the same dimensions, regardless of group composition and purpose. However, it does not clarify behavioral orientation. EPIT argues that these thoughts must be specifically interprofessional for an interprofessional identity to predict congruent behaviors. This principle is based on the identity-behavior congruence mechanism (30). The beliefs outlined by EPIT (19) and measured with EPIS (47) represent normative views on interprofessional collaboration, with individuals either agreeing or disagreeing with these perspectives. It is important to note that the EPIS subscale on interprofessional beliefs does not address perceptions of how specific clinical practitioners’ actions align with their specific professions. Instead, it focuses on a mindset that reflects how the individual believes professionals should behave. Consequently, to avoid cognitive dissonance, the individual is expected to act in accordance with these beliefs (48). In psychology, cognitive dissonance refers to the mental discomfort experienced when one’s beliefs and actions are inconsistent or contradictory. This discomfort often motivates a change in either beliefs or actions to achieve greater alignment and reduce the dissonance.

EPIS’s construct validity has been confirmed through Confirmatory Factor Analysis and this instrument demonstrates good to excellent internal consistency in the Netherlands (47), Lithuania (49), Germany (50), and Indonesia (51). However, the construct validity of Khalili’s Dual Identity Scale (31) and of Tong’s Interprofessional Three-Factor Model of Social Identity Scale (36) has yet to be confirmed by a confirmatory factor analysis.

## Interprofessional identity formation and activation according to EPIT

**Figure 3:**
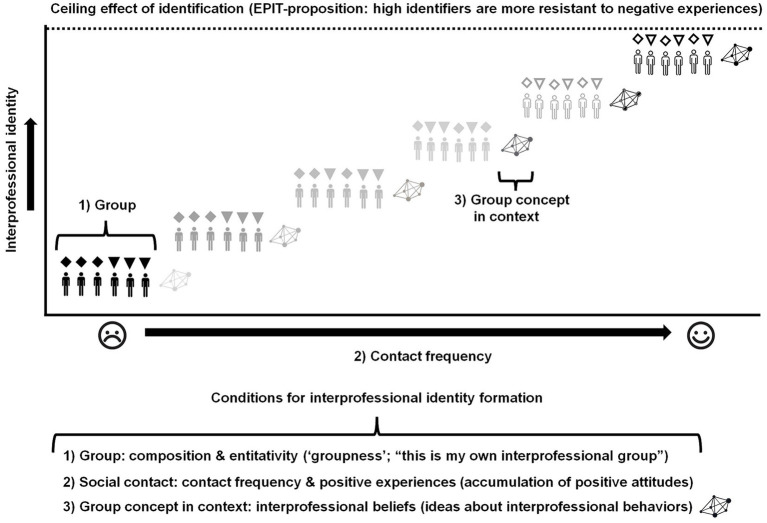
Interprofessional identity formation based on Extended Professional Identity Theory.

The formation of interprofessional identity is grounded in the principles of social identification with a specific group or social category. Traditionally, social identification in psychology is mostly measured by assessing one dimension, affective commitment (33, 34). This focus aligns with the primary purpose of most studies, which often centers on staff retention or career changes. Developing a three-dimensional instrument to measure a specific social identity poses a challenge. Beliefs or self-concepts related to a particular group, profession, or organization require custom-made measurement instruments, necessitating multiple psychometric studies to reliably assess the cognitions responsible for predicting unique congruent behaviors. This process is time-consuming, complex, and lacks feasibility because professions can change. However, interprofessional identity encompasses a definable set of beliefs related to interprofessional collaboration and is not confined to a single profession, which can be more dynamic, as demonstrated in Macdonald’s Sociology of Professions (52). Based on EPIT’s propositions, an interprofessional identity can be formed through a combination of three factors (Figure 3).

First, a group must be identifiable so that individuals can associate with it. If individuals do not seem connected, the collection will not be perceived as a social entity. This concept aligns with Campbell’s (53) theory of entitativity, which describes the degree of perceived “groupness.” This psychological perception is crucial for social identification, as individuals can only commit to an entity they recognize as existing. Thus, mere group composition is insufficient to create a social entity with which individuals can commit. For example, people waiting at a bus stop are usually a collection of unrelated individuals and are rarely perceived as a specific group. They form only a temporary queue that will disperse once each person has reached their destination. Entitativity is influenced by three factors: (1) similarity, (2) proximity, and (3) common fate. Enhancing entitativity can be achieved in several ways like intergroup comparison, emphasizing mixed profession group membership, or creating competition between such groups.

Second, contact frequency fosters a sense of belonging and commitment to a group or social category, provided these interactions are positive, leading to favorable attitudes (37, 38). Consequently, positive attitudes towards members of a mixed profession group should enhance interprofessional commitment (39). Interprofessional belonging as a member of a specific profession is likely to depend on professional beliefs related to the positioning of this profession in a larger community of various professions (54, 55). This positioning might be profession-centered but can also be more holistic. A more holistic positioning will make it more likely that interprofessional identity formation can be enhanced in a shorter time. Professional beliefs related to holistic professional positioning are likely to foster a stronger sense of interprofessional belonging, which is an aspect of interprofessional identity. Consequently, the formation of professional identity can partially influence the development of interprofessional identity. This is unrelated to the assumption that these two social identities are distinct social constructs.

A “ceiling effect” of interprofessional identification is necessary to cultivate a more robust interprofessional identity, which in turn fosters sustained motivation for interprofessional collaboration. This relies on a steady increase in interprofessional commitment, which is a form of affective commitment influenced by the frequency of positive social interactions (56). This also implies that interprofessional identification depends on the social proximity and interactions between members of various professions. Thus, the strength of an interprofessional identity is closely tied to the local social environment during a specific period. This suggests that relatively stronger (EPIT-based) interprofessional identities are likely to be more prevalent in secondary care compared to primary care, where various professions have less social interaction. A study on EPIT-based interprofessional identity among Dutch dietitians and physiotherapists working in primary care versus secondary care seems to support this assumption (57). When an intentional long-term strategy on interprofessional identity formation is applied, even individuals in primary care would probably be more prone to actively seek social contact with other professions and create interprofessional networks in primary care settings or beyond. This is based on the idea that interprofessional identity is a source of motivation towards interprofessional collaboration (58). Thus, a stronger interprofessional identity represents a stronger intention to initiate interprofessional collaboration (independent of competence and environmental factors). The degree of social cohesion within mixed profession groups should be enhanced by the affective commitment of individual mixed profession group members (18, 59). When this interprofessional commitment influences social cohesion, it will also improve psychological functioning by enhancing individual resilience (60). Resilience is the individual’s ability to withstand negative and hopeless situations when facing a problem (61). Resilient individuals are also more capable of making effective decisions under pressure compared to less resilient individuals (62).

Third, group concept is required to develop a self-concept as a group member. The relationship between group concept and group membership will shape the nature of social identity and, consequently, influence the behaviors exhibited when this identity is activated. In this case, an interprofessional identity. The self-concept related to an interprofessional identity is related to something that is accepted, considered to be true, or held as an opinion by the individual identifier (63). In other words, interprofessional beliefs as an identity dimension. These interprofessional beliefs will guide behavioral orientation linking interprofessional identity with congruent interprofessional behaviors (30).

Based on Tajfel (24) definition of social identity, supported by findings from Cameron (25) and Obst and White (26) and the confirmation of the three-dimensionality of EPIT-based interprofessional identity across four countries (47, 49–51), it is plausible to expect a degree of interprofessional identification even before actual interprofessional socialization. This phenomenon is referred to as “anticipatory socialization” (64). In other words, individuals can feel a sense of connectedness and hold certain beliefs prior to becoming group members. Thus, everyone can already possess an interprofessional identity, although it may be weak. This also depends on the degree of social contact with (future) members of other professions in (future) work-related situations. Therefore, it also depends on the social proximity of (future) members of other professions.

Students who infrequently encounter peers from other professions in contexts relevant to their future careers are likely to have a weaker interprofessional identity compared to those who regularly interact with students from other professions, even if these interactions are not directly interprofessional. However, it is unlikely that an interprofessional identity will be very strong before interprofessional socialization begins, as social identification is primarily strengthened through social contact. A pre-socialization interprofessional identification has been confirmed by a study conducted among dental and dental hygiene students in the Netherlands (58). Halfway through their studies but prior to their interprofessional education (IPE), Dutch dental and dental hygiene students in the city of Groningen exhibit a certain degree of interprofessional identification. However, the circumstances of these particular student groups differ from most curricula in other Dutch cities because they share the same facilities, such as a skills lab and student clinics. Since these students were accustomed to almost daily social proximity, they may have developed a stronger interprofessional commitment. However, since they did not participate in any IPE, they did not develop stronger interprofessional beliefs beforehand. Since interprofessional beliefs guide congruent interprofessional behaviors, interprofessional hierarchy was still present among dental and dental hygiene students without prior IPE experience but with close proximity (65).

As all three identity dimensions take time to develop, interprofessional identity formation during IPE requires a long-term strategy (33, 34). This approach ensures a more sustainable interprofessional identification by fostering higher interprofessional commitment, which, in turn, leads to increased motivation towards interprofessional collaboration (3, 58), combined with interprofessional belonging and beliefs when triggered by certain contextual cues (“identity triggers”). Without frequent interprofessional socialization, graduates may lose their motivation for interprofessional collaboration when faced with discouragement in their new workplaces, particularly if they feel excluded, have minimal interprofessional contact, or are not convinced that interprofessional collaboration can achieve desired results.

Drenth et al. (3) demonstrated the principles of EPIT-based interprofessional identity formation and its relation to team dynamics and outcomes in a rehabilitation setting. Within a rehabilitation center, they formed six mixed profession groups. These groups met regularly for 15 months during in-person and online sessions. During these sessions, work-related issues were discussed within the same mixed profession group, ensuring active and frequent engagement in a work-related context. Throughout these sessions, participants developed interprofessional beliefs by discussing shared values, organizational context and structure, group dynamics and interactions, as well as entrepreneurship and business management (66). The mixed profession groups could develop interprofessional belonging by their designated mixed profession group membership and interprofessional commitment by contact frequency. Interprofessional beliefs were cultivated by promoting a mindset that is psychologically linked to belonging to a mixed profession group. After this period, participants’ interprofessional identities significantly increased, group dynamics improved, and efficiency rose by 10–15%, related to an average decrease of almost 12 in-patient days while maintaining the same quality of care. Based on this study, interprofessional identity was associated with improved team dynamics and outcomes; however, the causal relationship between interprofessional identity and congruent behaviors was not established. Additional research is needed to investigate this causal relationship and explore the activation of interprofessional identity.

**Figure 4:**
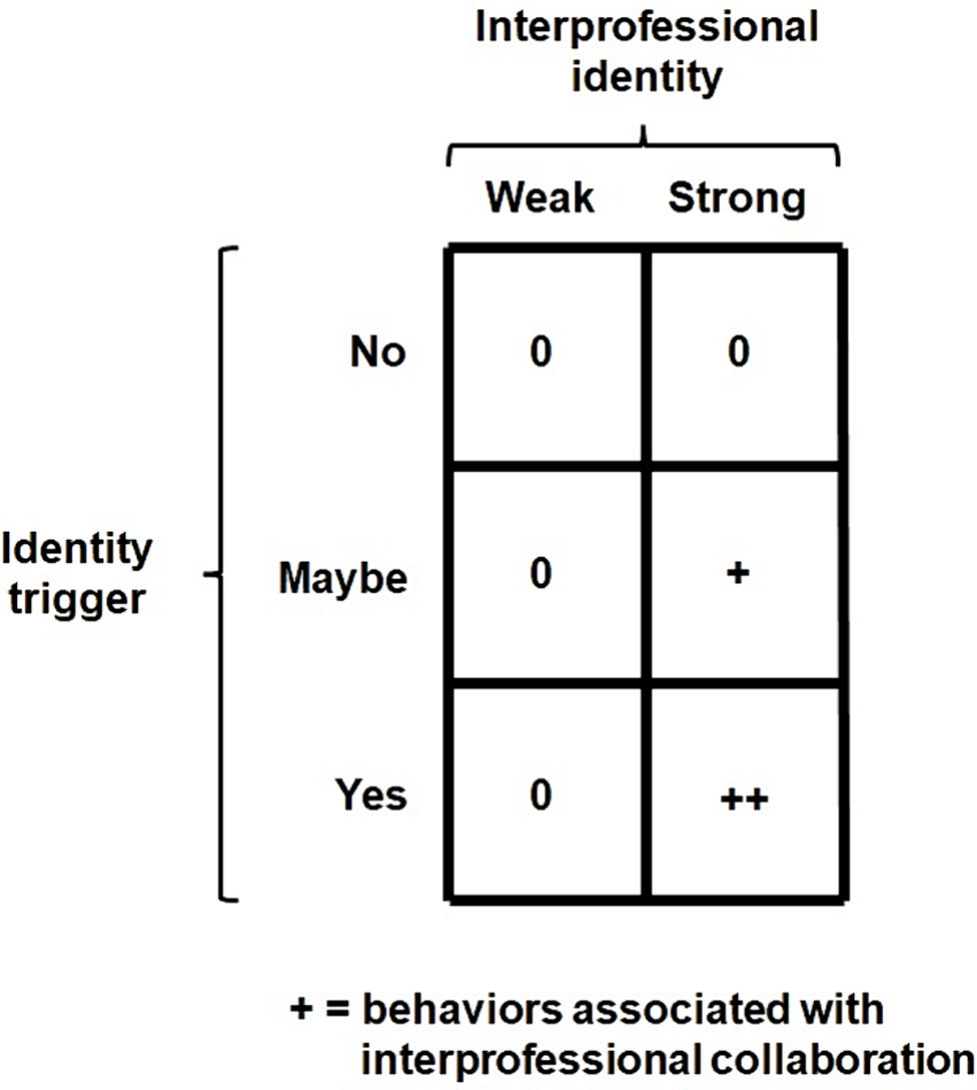
Interprofessional identity triggers.

The activation of an interprofessional identity only happens when the individual perceives a context with cues relevant for this social identity. Thus, when no contextual cues are recognized as interprofessional identity triggers, no congruent interprofessional behaviors will be displayed (Figure 4). This also implies that interprofessional identity triggers are learned and rely on knowledge as part of interprofessional competencies. Such competencies are acquired during IPE and workplace learning in practice.

Interprofessional identity should predict behaviors related to interprofessional collaboration. Which the WHO defines as “interprofessional collaboration occurs when health workers from various professional backgrounds collaborate with patients, families, carers, and communities to deliver the highest quality of care across settings” (67). Of course interprofessional collaboration is also required for shared problem domains outside and beyond the healthcare setting and can apply to other issues than health.

**Figure 5:**
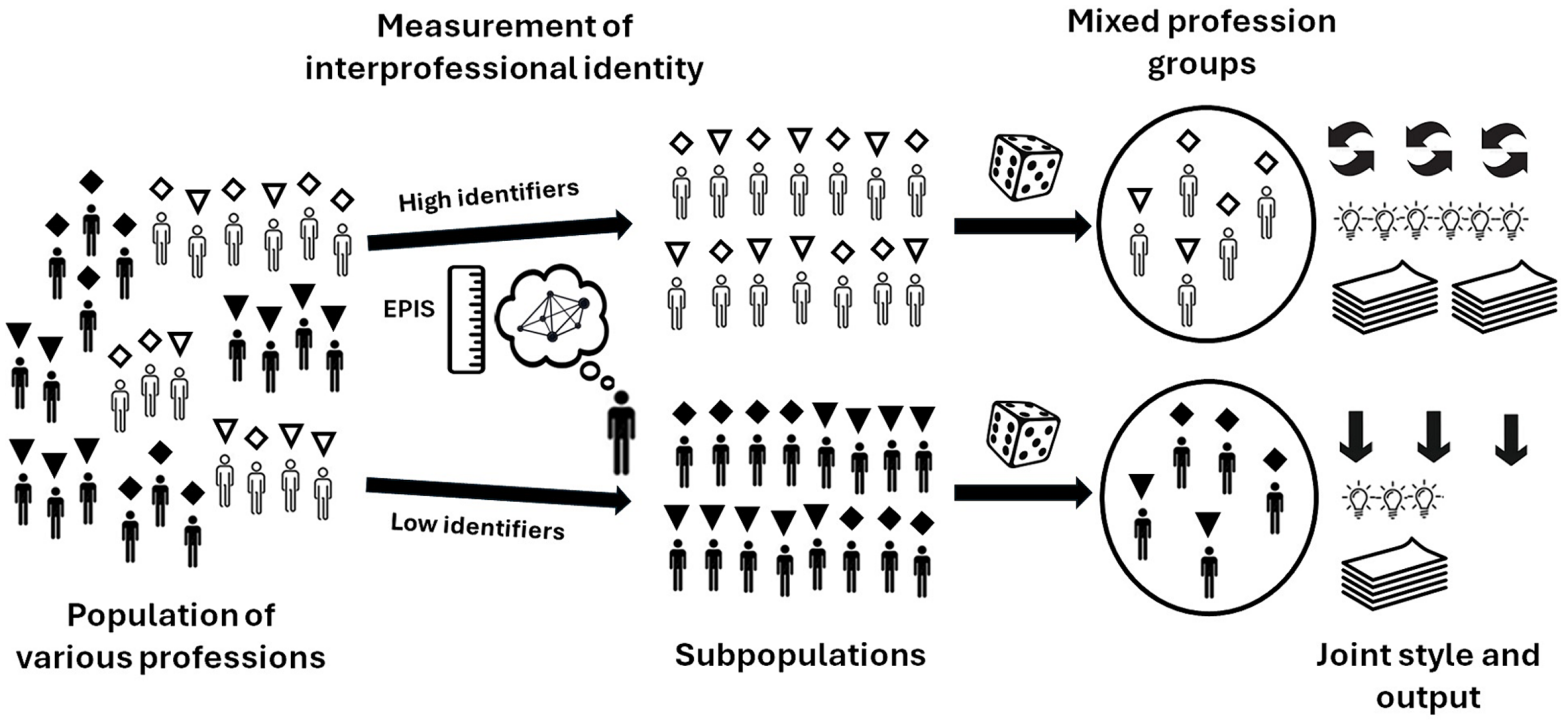
Interprofessional identity as an individual predictor of joint outcomes.

According to Tajfel (24), social identity acts as a source of motivation when activated by an identity trigger. Like motivation, social identity influences the intensity, direction, and persistence of an individual’s effort toward achieving a desired goal (68). The primary difference between social identity and motivation is the sense of belonging, which involves an internalization or psychological social association within the individual. Since interprofessional identity is a source of motivation for interprofessional collaboration, forming part of an individual’s self-concept and being triggered by contextual cues or identity triggers, the interprofessional identities of unrelated individuals should collectively predict outcomes related to shared problem domains. This also implies that individuals with a strong interprofessional identity would consistently function this way, regardless of their (new) teammates or network. Just like interprofessional competence, individuals carry their interprofessional identity with them wherever they work. To test this assumption, we measured EPIT-based interprofessional identity in a student population of dental and dental hygiene students before they participated in IPE and before they were a member of a mixed profession group (Figure 5).

After identifying individuals with strong interprofessional identities (high identifiers) and weak interprofessional identities (low identifiers) within each profession, they were randomly assigned to mixed profession groups under either strong or weak interprofessional identity conditions (58). The Extended Professional Identity Scale (EPIS) was used to measure interprofessional identity (47). Despite the relatively small difference in the degree of interprofessional identification between the two conditions, the difference was significant. Eight weeks after measuring their individual interprofessional identities, we presented the same problems to be solved by mixed profession groups in both conditions. We found that groups with relatively strong interprofessional identities performed better than those in the other condition. Students with strong interprofessional identities were more socially interactive within their own mixed profession group. These groups also generated more solutions to shared problems. A replication by an ongoing and unpublished study yields similar promising findings, showing the same patterns and indicating that greater differences in interprofessional identification between conditions also result in greater differences in joint outcomes.

## Showcasing EPIT-based interprofessional identity research and developments in Türkiye

**Figure 6:**
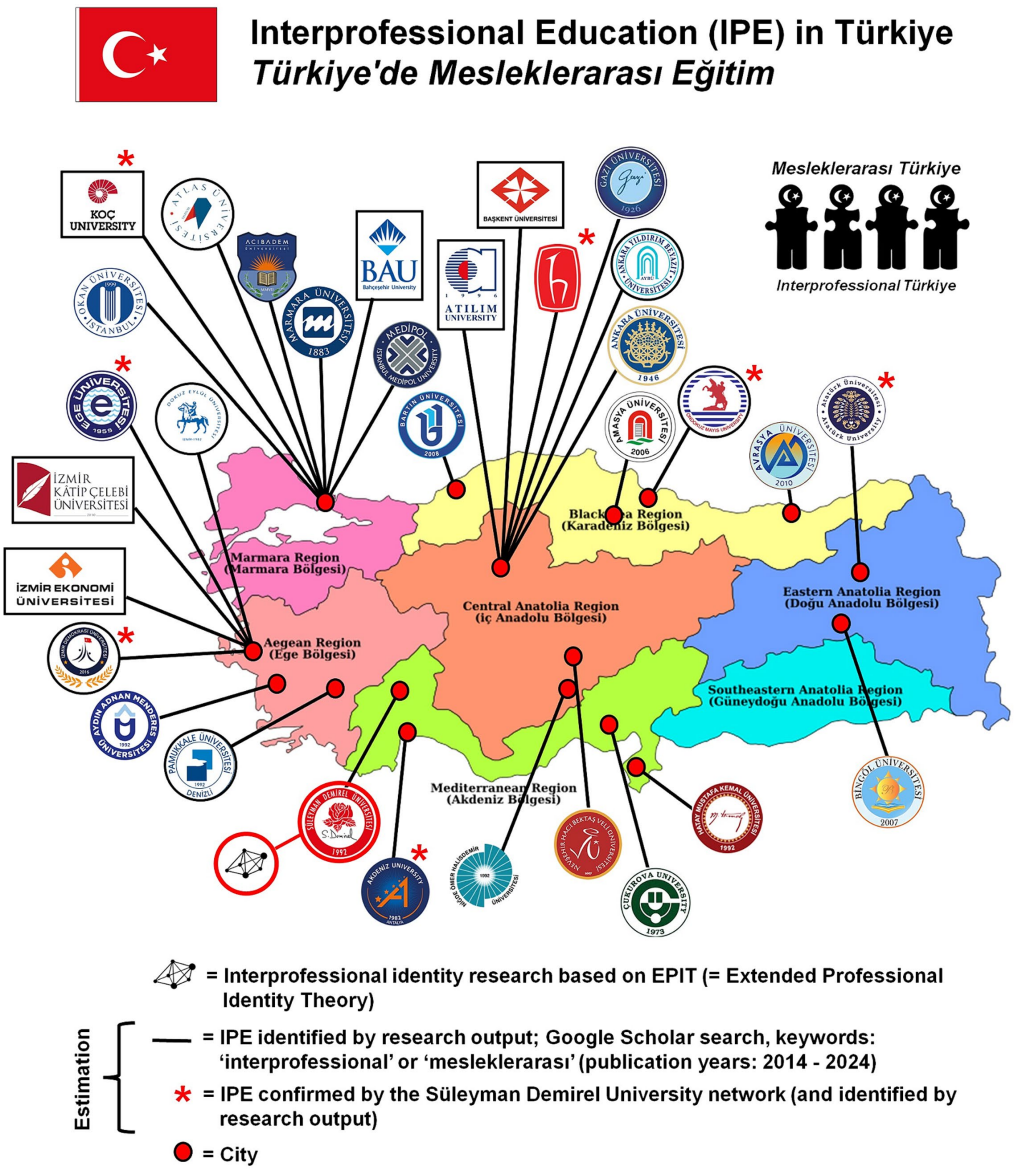
Turkish universities with interprofessional education or plans for its implementation.

In Türkiye, there are review studies on interprofessional education, cross-sectional descriptive, and experimental studies covering various professions, scale adaptation and scale development studies, program development studies, training activities independent of education programs such as interprofessional education academy and examples of trainer development programs (69–86). The inclusion of an article on interprofessional education in the accreditation standards for medical faculties, along with the emphasis on interprofessional communication and teamwork, is a significant step, even though these standards are not explicitly part of the accreditation for nursing, health sciences, and dentistry education. Nonetheless, many health professionals faculties have made it a goal to address these competencies in their curricula. As a result, courses focusing on interprofessional education have been incorporated into their curricula. These efforts are seen as crucial steps toward establishing the foundation required to enhance interprofessional collaboration in practice. The number of Turkish universities that are engaged in interprofessional education has increased considerably in the past 10 years (Figure 6). Google Scholar can provide some indication of interprofessional education developments in Türkiye based on national and international research output between 2014 and 2024. However, the number of Turkish universities conducting preparatory research on interprofessional education or evaluating their interprofessional education activities is currently relatively small. Out of 207 Turkish universities (87), only 15.5% (32 universities) appear to be engaged in interprofessional education. This percentage is only an estimation. Despite the relatively small number of Turkish institutions with IPE, the number of Turkish institutions involved in IPE research increased after 2019. The latter indicates a greater increase of Turkish IPE development and implementation compared to earlier years.

In addition to the growing emphasis on interprofessional education, collaborative practice is also on the rise. A study evaluating interprofessional collaboration within a real work environment in palliative care services at Tekirdağ Dr. İsmail Fehmi Cumalıoğlu City Hospital exemplifies this trend. Presented as an oral presentation at the 1st International Eastern Black Sea Family Medicine Congress, held from May 25–27, 2023, the research highlights the significance of collaborative efforts among healthcare professionals in improving the quality of palliative care (88). This study showcases practical applications and outcomes in a clinical setting, underlining the crucial role of teamwork in enhancing patient care.

An indication that interprofessional collaboration is gaining importance in Türkiye is the increasing development and adaptation of related measurement instruments. Numerous tools have been created to assess interprofessional education and collaboration. Many of these instruments, such as a Turkish interprofessional identity scale (EPIS-TR, the Turkish translation of EPIS), attitude scales (e.g., RIPLS and IPAS), and teamwork scales (e.g., SITAT), have already been completed, while many others are still in progress of development or publication (89, 90).

## Future directions for interprofessional identity research in the Turkish context

Numerous discussions at both international and national levels have centered on the challenges of evaluating the impact of interprofessional education and collaboration on health service delivery and outcomes. To address this issue, interprofessional research is being planned to offer diverse and comprehensive assessment opportunities. Many areas within this field remain scientifically unexplored and await investigation. This also includes interprofessional identity as a new research domain within interprofessional education and collaborative practice research.

Organizing training programs to enhance interprofessional collaboration at the local level presents significant challenges. In Türkiye, health-related professions typically follow discipline-based and integrated training programs. Integrating an additional program for a common educational purpose within these existing frameworks is highly complex. Therefore, establishing a university-wide unit called the “Interprofessional Education Coordinatorship,” which includes representatives from all health-related educational units, may be an appropriate solution. This organization would enable each representative to better align their individual programs with the joint interprofessional program, actively contributing to the development, implementation, and maintenance of the collaborative curriculum. Additionally, representatives from local “Interprofessional Education Coordinatorships” could form a national non-governmental organization dedicated to interprofessional education and collaboration. This organization would provide a platform for local coordinators to share best practices, find alternative solutions to common problems, and design new collaborations and scientific research initiatives. Such national structuring would facilitate the effective resolution of local issues through benchmarking, while also providing opportunities for publishing in scientific journals and organizing conferences and symposiums dedicated to interprofessional education, collaboration, and research. At Süleyman Demirel University, the working principles of the “Interprofessional Education Coordinatorship” have already been established, and efforts to further develop this unit are ongoing.

For interprofessional collaboration to effectively enhance the quality of national health services, it is crucial to ensure representation at the ministerial level. Having ministry-level representation focusing on interprofessional identity and competencies that directly improve health service delivery is invaluable for ensuring the smooth operation of this process. Collaborative efforts developed jointly with the Ministry of Health, universities, and non-governmental organizations will play a pivotal role in quality enhancement processes by fostering a culture of interprofessional collaboration nationwide. To advance this initiative, Süleyman Demirel University is spearheading the “1st Interprofessional Education Academy,” supported by TÜBİTAK for the first time in 2024. This national-level interprofessional training event will involve trainers from five Turkish universities (91).

While the context and necessity for interprofessional education and interprofessional identity and collaboration research are internationally recognized (92), addressing issues and devising solutions must be tailored to each nation’s specific circumstances. Therefore, despite national-level barriers and limitations, every country has the potential to contribute scientifically to this field. By fostering international collaboration among researchers and practitioners, countries can enrich the global knowledge base on interprofessional identity, education, and collaboration, leading to the identification of effective approaches to local challenges. For example, even though the measurement of EPIT-based interprofessional identity has similar psychometric properties in different countries (47, 49–51), the formation of interprofessional identity might depend on cultural differences. In addition, it is likely that the style of congruent behaviors predicted by interprofessional identity are different depending on culture due to different customs, habits and values. Also, it is likely that the overall degree of interprofessional identification will depend on social circumstances and norms in a country apart from the local social environment (57). Numerous organizations play pivotal leadership roles in advancing interprofessional education, collaboration, and research globally, actively striving to develop and expand this field (93, 94).

Global organizations for interprofessional education and collaborative practice share a common goal: to advance the theoretical foundations of interprofessional education and collaborative practice, address practical challenges, and propose solutions through international scientific and cultural exchanges in the domain of interprofessional education, collaboration, and research. Turkish scholars participate at various levels in these organizations. Although advancing interprofessional collaboration research in Türkiye may seem challenging, progress is promising through scientific inquiry and international engagement grounded in solid theoretical foundations. By consolidating these efforts into national-level organizations through local collaborations, Türkiye has the potential to assert itself more effectively on the global stage. This can be achieved by comparison and learning from local solutions such as measuring interprofessional identity, cultivating it, and enhancing interprofessional collaboration by systematically enhancing stronger interprofessional identities within the Turkish context.

## Data Availability

The original contributions presented in the study are included in the article/supplementary material, further inquiries can be directed to the corresponding author/s.
